# Effects of Chloride and Sulfate Salts on Seed Germination and Seedling Growth of *Ballota hirsuta* Benth. and *Myrtus communis* L.

**DOI:** 10.3390/plants12223906

**Published:** 2023-11-20

**Authors:** Mohammed Dadach, Muhammad Zaheer Ahmed, Arvind Bhatt, Emanuele Radicetti, Roberto Mancinelli

**Affiliations:** 1 Ecology and Environment Research Laboratory, Faculty of Nature and Life Sciences, Bejaia University, Targua Ouzemour, Bejaia 06000, Algeria; mohammed.dadach@univ-bejaia.dz; 2 Dr. M. Ajmal Khan-Institute of Sustainable Halophyte Utilization (MAK-ISHU), Faculty of Science, University of Karachi, Karachi 75270, Pakistan; mzahmed@uok.edu.pk; 3 Lushan Botanical Garden, Chinese Academy of Sciences, Jiujiang 332900, China; drbhatt79@gmail.com; 4 Department of Chemical, Pharmaceutical and Agricultural Sciences (DOCPAS), University of Ferrara, 44121 Ferrara, Italy; emanuele.radicetti@unife.it; 5 Department of Agricultural and Forestry Sciences (DAFNE), University of Tuscia, 01011 Viterbo, Italy

**Keywords:** seed germination, seedling growth, salt stress, medicinal plants, chloride, sulfate

## Abstract

Soil salinity is a well-known abiotic factor affecting the germination and seedling growth of various plant species. Therefore, we evaluated the effects of different chloride salts (NaCl, KCl and MgCl_2_) and sulfate salts (Na_2_SO_4_, K_2_SO_4_ and MgSO_4_) on the seed germination and early seedling growth of two important ethnomedicinal shrubs of North Africa and the Mediterranean basin (*Ballota hirsuta* and *Myrtus communis*). Seeds of these species were subjected to five salinity levels (0–100 mM) and incubated at 20 °C under a light regime (12 h photoperiod). Both species demonstrated their highest germination percentage under control conditions (i.e., without salinity). However, as salinity levels increased, the germination percentages for both species decreased, regardless of the type of salt used. Cations appeared to be more determinative than the anions in regulating the seed germination of both species. *M. communis* seeds displayed greater sensitivity to sodium (Na^+^) salts, especially when accompanied with chloride (Cl^−^) anions. At the higher salt concentrations (75 and 100 mM), Na^+^ salts had a more pronounced inhibitory effect on *M. communis* seedling growth compared to potassium (K^+^) and magnesium (Mg^2+^) salts. Conversely, Mg^2+^ salts were more detrimental to seedling growth in *B. hirsuta*. Based on our results, it can be concluded that both of these species are able to tolerate a moderate level of salinity. Overall, *B. hirsuta* may be a promising choice for rehabilitating the soils dominated by chloride salts, while *M. communis* could be utilized for restoring sulfate-dominated soils.

## 1. Introduction

Soil salinization has become a major global problem which negatively affects plant growth and establishment by interfering with various physiological processes due to osmotic stress and ionic imbalance [1]. Generally, salt-affected soils are categorized into three groups (i.e., saline, sodic and saline–sodic soils) based on the type and extent of salts present. Total soluble salts (measured by electrical conductivity, EC), water potential and pH are mainly used to characterize salt-contaminated soils [2]. Saline soils pose a significant challenge due to the prevalence of soluble salts, particularly sulfate (SO_4_^2−^) and chloride (Cl^−^) salts. Additionally, the adverse impact of salinity can be exacerbated when the soil contains a high concentration of sodium (Na^+^) [3]. High evaporation, which greatly exceeds precipitation in arid and semi-arid regions, leads to the accumulation of salts on the soil surface that results in irreversible alterations in the soil’s physicochemical properties and which ultimately hampers plant growth and survival [4,5].

Selection of salt-resistant species is considered to be the first and most important step for reclaiming salt-affected land. Therefore, understanding salinity tolerance at early growth phases (i.e., seed germination and seedling establishment stages) could be an effective screening method to identify suitable species for the phytoremediation of degraded saline lands [6]. Seed germination and seedling establishment are considered to be the most critical phases in a plant’s life cycle because they play a pivotal role in determining the species survival, especially under unfavorable conditions [7]. Salinity exerts a negative influence on seed germination by disrupting enzymatic activities, interfering with protein metabolism and impeding the mobilization of the seed’s reserves [8]. Moreover, the presence of excessive amounts of salt in soil decreases growth by compromising the ability of seedlings to take up water (osmotic stress) and/or altering cellular ion homeostasis (ionic stress). Excessive accumulation of Na^+^ and Cl^−^ in the plant tissues leads to severe ion imbalance that results in an alteration in the stomatal regulation, disruption of the chlorophyll biosynthesis and inactivation of plant growth hormones; all these circumstances subsequently affect seedling establishment [9,10].

Sodium chloride is widely used to test salinity tolerance during the seed germination and seedling establishment stages because of its prevalence in the inland and coastal soils of the arid and semi-arid regions [11]. However, sometimes many other ions such as K^+^, Mg^2+^, Ca^2+^, SO_4_^2−^ and CO_3_^2−^ may exist in higher quantities as compared to Na^+^ and Cl^−^ [12,13]. The presence of certain cations (i.e., Na^+^, K^+^, Ca^2+^ and Mg^2+^) and their accompanying anions (e.g., Cl^−^, NO_3_^−^ and SO_4_^2−^) also severely affects germination and plant development [14]. Furthermore, it has been documented that various salt agents may have differing effects during the germination and seedling establishment stages, and these effects can vary depending on the species. For example, *Lavandula stoechas* seeds exhibited better tolerance to MgCl_2_ compared to other dissolved salts [15]. On the other hand, *Artemisia herba-alba* seeds displayed greater susceptibility to MgCl_2_ when compared to NaCl, CaCl_2_ and Na_2_SO_4_ [6]. A similar trend in salt tolerance was observed for *Chenopodium glaucum* [16] and *Zygophyllum simplex* [17] during seed germination.

North Africa is suffering from frequent and prolonged drought events and thus it is considered to be the third most salt-affected region after the Middle East and Australia [18]. Saline soils in North African countries are primarily characterized by the presence of various chloride- and sulfate-based salts, although NaCl is the prominent soluble salt (>50%) in these countries. Moreover, the principal cation components in the North African soils are Na^+^ and Mg^2+^ [6]. The expansion and sustainability of plant populations depends on successful seed germination and subsequent seedling establishment. However, different salt types can have varying effects on plants. Therefore, we investigated the effects of different chloride and sulfate salts on the seed germination and seedling establishment of two medicinally important species.

*Ballota hirsuta* Benth. (Family of Lamiaceae) is a perennial, low-growing sub-shrub, native to the Mediterranean region. It is commonly found in a variety of habitats, including open forests, rangelands, plains, and low-elevation mountainous areas within the semi-arid zones of North Africa and southern Europe [19]. A previous study indicated that *B. hirsuta* seeds exhibited a remarkable germination capacity across a wide temperature range (5 to 30 °C) under controlled laboratory conditions. However, salinity (using NaCl) and drought stress (simulated by polyethylene glycol) severely restricted seed germination [20]. The flavonoids (viz., quercetin-3-glucodide and luteolin-7-rutinoside) found in this plant have been employed in the treatment of various health conditions [21]. In Algeria, this species is known as “*Merouit*” and is traditionally utilized for the treatment of bruises, injuries and rheumatic pain [22]. Recently, 43 essential oil compounds have been identified in *B. hirsuta*, rendering it a valuable candidate for cosmetic applications [23]. Harsh climatic conditions and anthropogenic pressures are the main factors disturbing various *B. hirsuta* populations in their natural habitat [24].

*Myrtus communis* L. (Family of Myrtaceae) is a small perennial tree, native to the Mediterranean region and North Africa and now widely naturalized in many regions, such as in South America, Northwestern Himalaya and Australia. This species is commonly found in coastal areas, inland hills and open fields, and its growth habits can vary from a small tree to a large shrub depending on environmental conditions, genetic plasticity and growth patterns [25,26]. Moreover, this species is regarded as a promising and adaptable species that could effectively fit into transitional production strategies on a large scale in moderately saline lands of the Mediterranean region [26]. In folk medicine, *M. communis* is employed for its antiseptic, anti-inflammatory, disinfectant and hypoglycemic properties and also used to cure many diseases such as lung disorder, stomach pains, cough and poor appetite [27]. Moreover, this species is very aromatic due to the high essential oil content in its leaves, and it is mainly utilized in the production of perfume and cosmetic products [28]. Evidence shows that various factors such as soil salinity, prolonged drought and land-clearing activities are severely affecting the growing area of *M. communis* [15].

The concept of large-scale cultivation of underutilized plants, particularly medicinal ones, as part of non-conventional agricultural practices, has emerged as an innovative and sustainable strategy. This approach aligns with the principles of promoting clean agro-biodiversity and ensuring food and nutrition security for disadvantaged and marginalized populations, especially in developing countries. Furthermore, implementing such practices in marginal land is reported to contribute to achieving various sustainable development goals, including crop diversification, improved health and well-being, decent employment, poverty reduction and the conservation of natural resources [29]. Consequently, promoting the propagation of neglected crops, including medicinal plants, stands as a critical measure to enhance nutritional security and ecological resilience while minimizing adverse effects on food production.

Studies examining the impact of various salts on seed germination and seedling establishment, particularly concerning medicinal plants native to North Africa and the Mediterranean region, have been relatively underexplored despite their significant importance [15,30]. Therefore, the aim of this study was to examine the effects of chloride salts (NaCl, KCl and MgCl_2_) and sulfate salts (Na_2_SO_4_, K_2_SO_4_ and MgSO_4_) on seed germination and seedling establishment of *B. hirsuta* and *M. communis*. The outcome of this investigation will lead to understanding of the innate response of these species during their early life phases when they face salinity, and hence could be used for optimizing their cultivation in salt-affected soils.

## 2. Results

### 2.1. Salt Stress Effect on Germination Parameters 

The analysis of variance (ANOVA) indicated a significant effect of salt types (S) and salt concentrations (SCs) on all seed germination attributes of *B. hirsuta*, with the exception of the synchrony index (Syn), which was not affected (*p* > 0.05). Notably, the interaction between S and SC significantly influenced the final germination percentage (FGP), decreasing germination percentage (DGP) and uncertainty (Unc) (Table 1). A similar pattern was observed in the case of *M. communis*, with the exception of Unc. In this species, the interaction between S and SCs did not show a significant effect (Table 2).

Both species, *B. hirsuta* and *M. communis*, exhibited their highest germination percentages under control conditions (without salinity), achieving 85% and 90%, respectively (Table 3 and Table 4). The seed germination percentage of both the species decreased with increasing salinity concentration, irrespective of S. However, the effects of different types of salts on the seed germination appeared to be species-specific. Specifically, *M. communis* consistently maintained a germination percentage exceeding 14% at a concentration of 100 mM, regardless of the S. In contrast, none of the *B. hirsuta* seeds were able to germinate at 100 mM when exposed to K_2_SO_4_, MgSO_4_ and MgCl_2_ (Table 3).

Germination percentage of *M. communis* seeds was lower than 50% at 75 mM (DGP < 50%) under all the tested S. However, at 100 mM, the decline in germination percentage followed the order MgSO_4_ > MgCl_2_ > Na_2_SO_4_ = KCl = K_2_SO_4_ > NaCl. In the case of *B. hirsuta*, a germination percentage of less than 50% (DGP < 50%) was observed prior to reaching 75 mM for Na_2_SO_4_, K_2_SO_4_ and MgCl_2_. At 100 mM, the decline in germination percentage followed this order NaCl > Na_2_SO_4_ > KCl > K_2_SO_4_ = MgCl_2_ = MgSO_4_.

Salt stress extended the time of 50% germination (T_50_) and reduced germination speed (GSP) in both species, although a significant (*p <* 0.05) increase in mean germination time (MGT) was found only in the case of *M. communis* (Table 3 and Table 4). The exposure of seeds to 100 mM of different Cl^−^ and SO_4_^2−^ salts significantly (*p* < 0.05) reduced the Unc, except with NaCl and KCl for *B. hirsuta* and MgSO_4_ for *M. communis*. The Syn was higher for *B. hirsuta* as compared to *M. communis*, although Syn appeared to be unaffected by salinity concentration (Table 3 and Table 4).

**Figure 1:**
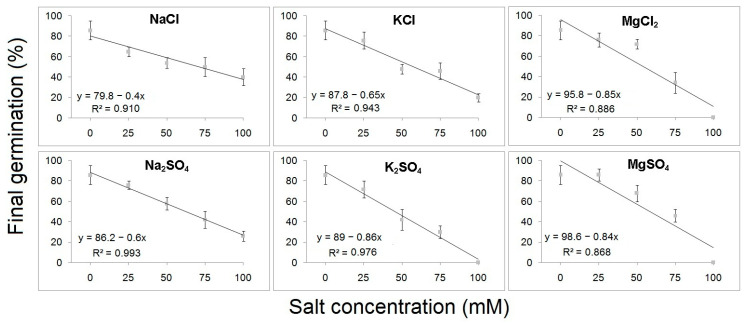
Linear regression analysis of final germination percentages of *B. hirsuta* seeds treated with different concentrations of chloride and sulfate salts.

**Figure 2:**
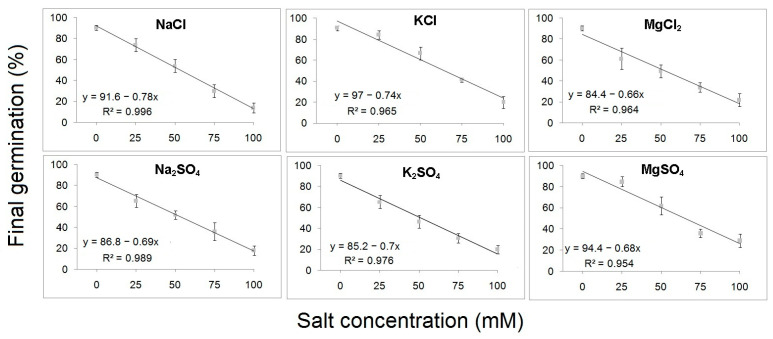
Linear regression analysis of final germination percentages of *M. communis* seeds treated with different concentrations of chloride and sulfate salts.

The coefficient of determination (R^2^) displayed a strong correlation between FGP and S with linear regression values ranging between 0.993 and 0.868 for *B. hirsuta* and 0.996 to 0.954 for *M. communis*. The slope of regression line was −0.86 and −0.78 in the case of seeds treated with K_2_SO_4_ and NaCl for *B. hirsuta* and *M. communis*, respectively, indicating that the maximum reduction in germination percentage with each 1 mM increase in salt was approximately 0.86% for *B. hirsuta* and 0.78% for *M. communis*, respectively (Figure 1 and Figure 2).

### 2.2. Salt Stress Effect on Seedling Growth

Seedling growth traits of *B. hirsuta* were significantly (*p* < 0.05) affected by S and SC. The two-way ANOVA analysis confirmed that the interaction of S and SC had a significant (*p* < 0.05) effect on hypocotyl length (HL), radicle length (RL), seedling tolerance index (STI) and seedling vigor index (SVI) (Table 1). A similar pattern was observed for *M. communis*, with the exception of RL and STI (Table 2). 

Increasing SC significantly decreased the seedling growth (both HL and RL), salinity tolerance and vigor indexes in both the species, except in *B. hirsuta*, where the low concentration (25 mM) of K^+^ and Mg^2+^ salts (either with Cl^−^ or SO_4_^2−^) improved HL only (>3 cm as compared to the control 2.96 cm) (Table 3). Hypocotyl growth of *M. communis* was severely inhibited in Na_2_SO_4_ (0.25 cm at 100 mM vs. 1.50 cm in the control), while the root growth was severely inhibited in K_2_SO_4_ (0.32 cm at 100 mM vs.1.26 cm in the control) (Table 4). However, seedling growth (both HL and RL) of *B. hirsuta* decreased in the following order of salts KCl > NaCl > Na_2_SO_4_ > K_2_SO_4_ > MgCl_2_ > MgSO_4_. 

## 3. Discussion

Understanding the effect of salinity during germination and seedling establishment is hypothetically essential for screening salt-resistant species that could be used for developing an effective cultivation strategy in areas affected by salinity. Encouraging the cultivation of medicinally important species is a plausible practice that will contribute to (1) reducing the pressure on wild populations, (2) assisting conservation actions, (3) meeting the demands of therapeutic uses and (4) increasing local peoples’ livelihoods [31,32].

Soil salinization is one of the major problems in North African and Mediterranean regions. Therefore, emphasis should be placed on restoring the native vegetation [33]. Selecting salt-resistant native plants with substantial economic and ecological potential could be among the innovative techniques to utilize these species for restoration/rehabilitation purposes.

In this study, *B. hirsuta* and *M. communis* showed a remarkable tolerance to different kinds of chloride- and sulfate salts during the germination stage. Seeds of both species were able to germinate even at 100 mM salinity, with the exceptions being MgCl_2_, K_2_SO_4_ and MgSO_4_ for *B. hirsuta* seeds. This level of tolerance during the germination stage is higher than the salinity tolerance capacity reported in the majority of glycophytes [34]. These findings are consistent with previous studies conducted on different Mediterranean glycophytic species such as *Artemisia herba–alba* [6], *Marrubium vulgare* [30], *L. stoechas* [15] and *Cistus monspeliensis* [35]. Generally, both the glycophytes and halophytes exhibited optimum germination in the absence of salt [36,37,38,39]. However, the suppressive effects of chloride and sulfate salts are less detrimental to the germination of halophytes [11].

Overall, increasing salinity levels hinder the germination capacity and homogeneity (i.e., increase asynchronized germination) in glycophytes and halophytes. In the present study, exposing seeds to different types of salts adversely affected the germination speed, with the slowest germination speed seen at the highest salinity level. These results are consistent with a previous study, in which NaCl, CaCl_2_, MgCl_2_ and Na_2_SO_4_ were found to significantly delay germination speed in *M. vulgare* [30]. Similarly, reductions in germination percentage and germination speed have been shown in *Zygophyllum album* seeds when treated with different levels (0 to 400 mM) of NaCl, CaCl_2_ and Na_2_SO_4_ [40]. Usually, seeds tend to maintain dormancy under salinity, which in turn contributes to reducing germination speed as well as germination percentage [41]. Delaying the germination under salinity conditions could be a bet-hedging strategy that may allow these seeds to remain persistent (i.e., fail to germinate while still viable) in soil seed banks until the occurrence of precipitation events that dilute the salinity and create suitable opportunities for germination and seedling establishment, especially in arid and semi-arid bio-climates [13,42]. Likewise, decreased germination homogeneity under salinity implies that seeds extend their germination period over time, thereby increasing the likelihood of some seedlings surviving challenging conditions (such as drought and salinity). This is in contrast to higher germination homogeneity, which leads to a simultaneous emergence of the entire seedling cohort.

Chloride appears to have a more pronounced inhibitory effect on the germination of *M. communis* compared to sulfate. Conversely, *B. hirsuta* seeds exhibited the opposite response. Previous studies revealed that Cl^−^ is metabolically more toxic than SO_4_^2−^ during seed germination in various species such as *Haloxylon ammodendron*, *Halogeton glomeratus* and *Lepidium latifolium* [11,12]. Usually, seed cells can easily uptake Cl^−^ either passively (following electrochemical potential gradient) or actively (via symporters or antiporters) that eventually contribute to hampering the germination fitness [43]. Moreover, chloride’s strong capacity to destabilize and alter the structure of primary and secondary metabolites, resulting in reduced osmotropic properties, leads to a subsequent accumulation of reactive oxygen species [44]. The toxicity of chloride arises via interference with its nutritive roles [45]; however, most of the Cl^−^ targeted molecules are still unknown and thus deserve further investigation. The higher seed germination of *B. hirsuta* in the presence of Cl^−^ may be attributed to the role of this anion in facilitating water uptake, which is crucial for successful radicle emergence. In our case, Cl^−^ salts appeared to be less inhibitory in both species at the seedling growth phase, which might be attributed to the role of Cl^−^ as a micronutrient [46], especially in the oxygen-evolving complex system of photosynthesis [47]. Sulfate is an essential macronutrient with different functions in plant metabolism and cell homeostasis. Sulfate is present in a reduced form in amino acids, lipids and proteins [48]. The assimilation of sulfate in plants occurs through H^+^/SO_4_^2−^ cotransporters [49] and is facilitated by adenosine-5′-phosphosulfate reductase [50]. In the present study, it is likely that the germination of *B. hirsuta* was influenced by the presence of accompanying cations, which reduced the solubility of SO_4_^2−^ in water [51].

The effects of cations on seed germination and seedling growth were species-specific, depending on both the type of cation and species. For example, *B. hirsuta* seeds exhibited a reduction in germination percentage in the following order of cations: Na^+^ > K^+^ > Mg^2+^, whereas *M. communis* seed germination declined in the order of Mg^2+^ > K^+^ > Na^+^. A similar toxic effect of Mg^2+^ was observed during the germination of *H. glomeratus, L. latifolium* and *Peganum harmala* seeds [11], while Na^+^ showed a severe toxic effect during the germination of *Chenopodium quinoa* [52] and *L. stoechas* seeds [15]. We hypothesize that seed germination is primarily influenced by cations due to their regulatory roles in various processes of metabolism, as well as their ability to interact with various stress-responsive signaling molecules [53]. For example, Mg^2+^ is one of the essential mesoelements that play a pivotal role in various physiological functions within plants. It interferes with a wide range of post-germination processes (e.g., chlorophyll biosynthesis and carbon fixation) due to its interrelated roles (i.e., cofactor and activator of a number of enzymes) [54]. However, the precise mechanisms by which Mg^2+^ regulates seed germination remain unclear. Both deficiency and excess of Mg^2+^ can induce stress and cause a disturbance in enzymes associated with carbohydrate metabolism, such as ructose-1,6-bisphosphatase and GDPD-glucose pyrophosphorylase [55]. Myrtle seeds are reported to contain fatty acids [56]. The higher tolerance of *M. communis* seeds to Mg^2+^ is presumably related to the role of this ion in stimulating lipase activity, particularly in high-oil seeds [11]. Consequently, myrtle oily seeds exhibited better performance in the presence of MgCl_2_ and MgSO_4_ in the growth medium.

Seeds subjected to higher doses of salts (75 and 100 mM) exhibited remarkable detrimental effects on seedling growth capacity. However, seeds treated with 25 mM did not show any toxic effects on seedling growth (i.e., high hypocotyl and radical lengths). The majority of dissolved salts, except NaCl and Na_2_SO_4_, demonstrated a promotion of *B. hirsuta* seedling growth at 25 mM. This growth promotion at the lowest concentration could be attributed to magnesium and potassium, which serve as two essential macronutrients involved in various physiological functions. For example, Mg^2+^ is a crucial divalent ion that plays a key role in stomatal movement (opening/closing), regulation of key photosynthetic enzymes and modulation of protein biosynthesis [54]. On the other hand, K^+^ is required by plants to accomplish many vital functions, such as (i) transport of organic metabolites, (ii) protein synthesis, (iii) maintenance of appropriate cellular pH, (iv) regulation of osmotic balance and (v) cell extension [57]. Moreover, *B. hirsuta* and *M. communis* seeds/seedlings exhibited a relatively high tolerance to Na^+^ salts, indicating their adaptability to cope with this potentially toxic ion. The capacity for rapid and robust seedling growth under high osmotic potential are beneficial adaptive characteristics that enable the development of a deep root system, allowing these species to access deeper soil horizons, thus increasing the likelihood of seedling survival even in the presence of salt.

## 4. Materials and Methods

### 4.1. Seed Harvesting Site

**Figure 3:**
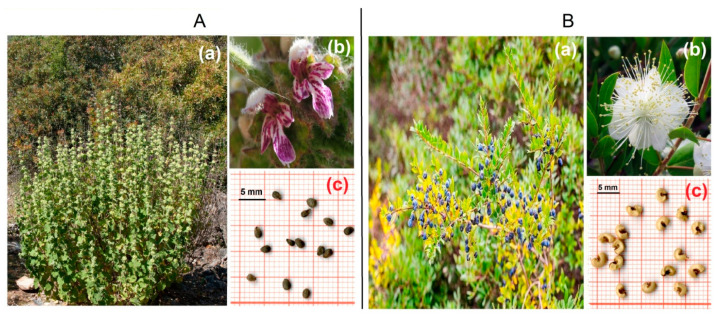
(**A**) Plant of *Ballota hirsuta* (**a**), flower (**b**) and seeds (**c**); (**B**) plant of *Myrtus communis* (**a**), flower (**b**) and seeds (**c**).

**Figure 4:**
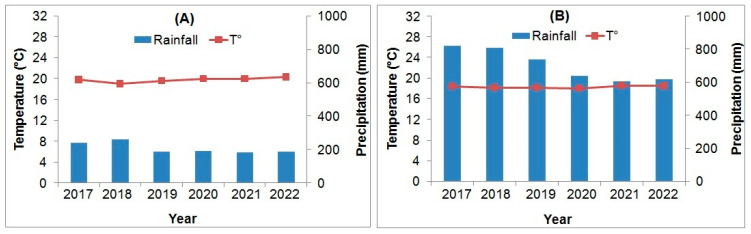
Last six-year climatic data (annual precipitations and mean temperatures) of Sidi Bel Abbes (**A**) and Béjaia (**B**) provinces (Source—Algerian National Meteorological office).

Mature seeds of *B. hirsuta* (Figure 3A) were collected during their natural dispersal in July 2022 from a naturally growing population situated near Sidi Bel Abbes city in Algeria (34°57′44.42″ N, 0°51′48.88″ E; 683 m above sea level). The seed collection site experiences a semi-arid climate with annual mean thermal amplitude of ≈20 °C (2.5 °C (minimum) and 35.5 °C (maximum)). Precipitation is erratic and limited (<220 mm per annum), occurring mostly during winter (Figure 4A). 

The ripe berries (blue-black capsules) of *M. communis* (Figure 3B) were collected in October 2021 from a naturally growing population located near Béjaia city in Algeria (36°44′54.07″ N, 5°01′23.67″ E; 73 m above sea level). This area is characterized by a sub-humid climate, with annual rainfall slightly exceeding 600 mm and an average temperature of about 18.5 °C (Figure 4B). Precipitation mainly occurs during autumn and winter. 

After being brought to the laboratory, the seeds were manually separated from the fruits, air-dried and subsequently stored in brown paper bags at 20 °C until the start of the experiment (ca. 3 months after collection).

### 4.2. Effect of Different Salts on Seed Germination

Seeds with uniform size, color and shape were used for the germination trials. To break both physical and physiological dormancy in *M. communis* seeds, they were subjected to a 1-month stratification period at 4 °C, followed by soaking in 250 ppm GA_3_ for 24 h [58]. Seeds of both species were surface-sterilized using 0.50% sodium hypochlorite for 10 min and subsequently washed thrice with deionized water to avoid fungal attacks. Seeds were sown in 9 cm Petri dishes lined with two disks of filter paper (Whatman No. 1) and moistened with 7 mL of different concentrations (0, 25, 50, 75 and 100 mM) of chloride (NaCl, KCl and MgCl_2_) and sulfate (Na_2_SO_4_, K_2_SO_4_ and MgSO_4_) salts. These concentrations were selected based on preliminary experiments on these species [20,26]. Four replicates of 25 seeds each were used for each treatment. Petri dishes were sealed with parafilm and placed in a seed incubator set at a constant temperature of 20 °C and 12 h photoperiod. The incubator was fitted with cool-white fluorescent tubes (≈25 µmol m^−2^ s^−1^); optimal conditions for the germination for most of the Mediterranean species [59]. Germinated seeds were counted every 48 h up to the 30-day period. Seeds were considered germinated with the emergence of the radicle (≥2 mm) [60].

### 4.3. Determination of Seed Germination Attributes 

Germination data were used to determine the final germination percentage (FGP), mean germination time (MGT), germination speed (GSP), uncertainty (Unc) and synchrony (Syn), as described by Lozano-Isla et al. [61]:

(1) $$FGP=\frac{Final\;portion\;of\;emerged\;seeds}{Total\;number\;of\;seeds}\times 100$$

(2) $$MGT=\frac{\sum_{i=1}^{k}t_{i}n_{i}}{\sum_{i=1}^{k}n_{i}}\times 100$$

(3) $$GSP=\frac{\sum_{i=1}^{k}n_{i}}{\sum_{i=1}^{k}n_{i}X_{i}}\times 100$$

(4) $$Unc=-\sum_{i=1}^{k}f_{i}{log}_{2}f_{i},\;\mathrm{being}\;f_{i}=\frac{n_{i}}{\sum_{i=1}^{k}n_{i}}$$

(5) $$Syn=\frac{\sum C_{n_{i},2}}{N},\;\mathrm{being}\;c_{n_{i},2}=\frac{n_{i}(n_{i}-1)}{2}\;\mathrm{and}\;N=\frac{\sum n_{i}(\sum n_{i}-1)}{2}$$

where *n_i_* is the number of seeds germinated at the *i*th time, *t_i_* is the number of days from the beginning of the germination test to the *i*th observation, *X_i_* is the period of germination experiments, *k* is the final day that germination was scored, *f_i_* is the relative germination frequency and *Cn_i,_*_2_ is the number of seeds germinated at the *i*th time interval. 

Decline in germination percentage (DGP) was calculated with the following equation [62]:

(6) $$DGP=\frac{FGP\;in\;control-FGP\;in\;stressful\;condition}{FGP\;in\;control}\times 100$$

Lower DGP values indicate higher salt resistance.

The time of 50% germination (T_50_) was determined using the following formula [63]:

(7) $$T_{50}=T_{i}+\frac{\left(\frac{N}{2}-N_{i}){(T}_{j}-T_{i}\right)}{N_{j}-N_{i}}$$

*N* is the total number of emerged seeds, and *T_i_* and *T_j_* are the time which seeds took to reach the adjacent counts of *N_i_* and *N_j_*, respectively, where *N_i_* < *N*/2 < *N_j_.*

The definition of each seed germination trait, as reported by Lozano-Isla et al. [61] and Ranal and Santana [64], are provided in Table 5.

### 4.4. Determination of Seedling Growth Parameters 

From each Petri plate, a random selection of five seedlings was sampled to evaluate growth parameters. The lengths of hypocotyls and radicles were individually measured at the end of germination experiments (after 30 days) using a graduated ruler. 

The seedling tolerance index (STI) was determined using the following equation [65]:

(8) $$STI=\frac{mean\;radicle\;length\;in\;stressful\;condition}{mean\;radicle\;length\;in\;distilled\;water}\times 100$$

Seedling vigor index (SVI) was calculated by using the formula suggested by Abdul-Baki and Anderson [66]:

(9) $$SVI=\left(mean\;hypocothyl\;length+mean\;radicle\;length\right)\times 100$$

### 4.5. Statistical Analysis

Germination and seedling growth data underwent arcsine transformation to ensure homogeneity of variance before performing an ANOVA. A two-way ANOVA was used to determine the differences among the effects of S, SC and their interaction on various dependent variables related to seed germination (FGP, MGT, GSP, Unc, Syn, DGP, T_50_) and seedling growth (HL, RL, STI and SVI). Subsequently, a pairwise *Tukey*’s (HSD) post hoc test (*p* ≤ 0.05) was conducted to determine the significant differences between means. Statistical analyses were carried out using IBM SPSS Statistics (version 22.0, SPSS Inc., Chicago, IL, USA). Additionally, linear regression analysis was performed to evaluate the coefficient of determination (R^2^) between the FGPs and different SCs for both the tested species.

## 5. Conclusions

We investigated the impact of different chloride (NaCl, KCl and MgCl_2_) and sulfate (Na_2_SO_4_, K_2_SO_4_, MgSO_4_) salts on the seed germination of *B. hirsuta* and *M. communis*. Despite the negative effects of salt stress, both species demonstrated resilience by successfully germinating even under higher salinity levels, reaching up to 75 mM. Chloride-based salts significantly inhibited the seed germination of *M. communis* compared to sulfate salts, while the response in *B. hirsuta* was the opposite. Milder salt concentrations, particularly those composed of Mg^2+^ and K^+^ at 25 mM, promoted seedling growth in *B. hirsuta.* Conversely, seedling growth in *M. communis* was more hindered by Na^+^ salts compared to K^+^ and Mg^2+^ salts. In general, chloride salts of Na^+^ and K^+^ were found to be less inhibitory than sulfate salts, while MgCl_2_ exhibited greater toxicity than MgSO_4_ for both of our studied species. Based on our findings, we proposed that both hold promise as salt-resistant glycophytes, making them valuable candidates for enhancing livelihoods and contributing to the restoration of North African and Mediterranean saline lands. 

## Figures and Tables

**Table 1:** Results of two-way ANOVA (mean squares, *F* ratio and *p* value) testing the influences of salts (S), concentrations (SCs) and their interactive effect (S × SC) on seed germination patterns and early seedling growth of *B. hirsuta*.

	Source of Variation								
Dependent Variables	Salt (S)			Concentration (SC)			S × SC		
	Mean Squares	*F* Ratio	*p* Value	Mean Squares	*F* Ratio	*p* Value	Mean Squares	*F* Ratio	*p* Value
FGP	354.74	4.338	0.002	14,172.71	173.31	0.000	341.14	4.17	0.000
DGP	501.90	3.27	0.011	19,472.32	196.94	0.000	479.94	3.14	0.000
MGT	0.58	5.62	0.000	1.37	13.29	0.000	0.15	1.44	0.153
GSP	87.34	7.38	0.000	170.02	14.36	0.000	18.52	1.56	0.108
T_50_	2.03	3.18	0.014	12.50	19.60	0.000	0.71	1.10	0.374
Unc	0.48	5.94	0.000	2.30	28.76	0.000	0.23	2.85	0.001
Syn	0.03	1.58	0.180	0.05	1.79	0.085	0.02	0.88	0.605
HL	6.58	41.10	0.000	23.20	144.99	0.000	1.12	6.98	0.000
RL	7.44	38.38	0.000	66.68	344.42	0.000	0.96	4.93	0.000
STI	2740.16	35.04	0.000	24,127.54	308.50	0.000	347.50	4.44	0.000
SVI	47,780.34	14.78	0.000	1,449,784.25	448.50	0.000	9273.34	2.86	0.001

FGP: final germination percentage; DGP: decreasing germination percentage; MGT: mean germination time; GSP: germination speed; T_50_: time to reach 50% of final germination; Unc: uncertainty; Syn: synchrony; HL: hypocotyl length; RL: radicle length; STI: seedling tolerance index; SVI: seedling vigor index.

**Table 2:** Results of two-way ANOVA (mean squares, *F* ratio and *p* value) testing the influences of soluble salts (S), concentrations (SCs) and their interactive effect (S × SC) on seed germination patterns and early seedling growth of *M. communis*.

	Source of Variation								
Dependent Variables	Salt (S)			Concentration (SC)			S × SC		
	Mean Squares	*F* Ratio	*p* Value	Mean squares	*F* Ratio	*p* Value	Mean Squares	*F* Ratio	*p* Value
FGP	14,224.70	437.24	0.000	316.90	9.74	0.000	78.15	2.40	0.005
DGP	17,320.08	409.80	0.000	387.19	9.16	0.000	96.40	2.28	0.007
MGT	23.24	57.70	0.000	1.53	3.80	0.005	0.52	1.30	0.215
GSP	90.74	80.76	0.000	5.86	5.22	0.000	1.75	1.56	0.094
T_50_	90.56	32.60	0.000	17.22	6.20	0.000	3.84	1.38	0.166
Unc	2.43	34.12	0.000	0.19	2.66	0.031	0.04	0.60	0.894
Syn	0.03	6.64	0.000	0.01	1.05	0.396	0.00	0.44	0.978
HL	3.02	65.18	0.000	0.5	10.84	0.000	0.10	2.20	0.010
RL	1.54	30.01	0.000	0.25	4.78	0.001	0.06	1.07	0.399
STI	8531.34	11.38	0.000	1849.84	2.46	0.042	391.58	0.52	0.946
SVI	154,656.5	198.00	0.000	8836.16	11.32	0.000	1921.54	2.46	0.004

FGP: final germination percentage; DGP: decreasing germination percentage; MGT: mean germination time; GSP: germination speed; T_50_: time to reach 50% of final germination; Unc: uncertainty; Syn: synchrony; HL: hypocotyl length; RL: radicle length; STI: seedling tolerance index; SVI: seedling vigor index.

**Table 3:** Seed germination patterns and seedling growth features of *B. hirsuta* in response to different concentrations of chloride- and sulfate-based salts.

Salts	SC (mM)	FGP (%)	DGP (%)	MGT (Days)	GSP (%)	T_50_ (Days)	Unc	Syn (bit)	HL (cm)	RL (cm)	STI	SVI
Control	0	85a	0a	2.46a	40.75a	3.22a	1.20a	0.50a	2.96ab	5.25a	100a	770.80a
NaCl	25	65b	23.52b	2.44a	41.42a	3.58a	1.14a	0.52a	2.74ab	4.34a	82.74a	460.50b
	50	54b	36.47c	2.72a	37.15a	4.10ab	1.30a	0.40a	2.30ab	3.00b	57.06b	284.68c
	75	54b	36.47c	3.02a	33.47ab	4.80ab	1.56a	0.32a	1.74bc	2.06b	39.28c	209.50d
	100	40c	52.94c	3.08a	33.25ab	5.00b	0.96ab	0.30a	0.62c	0.58cd	11.02d	48.58e
KCl	25	78a	8.24ab	2.45a	40.85a	3.45a	1.18a	0.50a	3.56a	3.68ab	70.56ab	560.62ab
	50	48bc	45.88c	2.55a	39.77a	3.76a	1.16a	0.50a	3.22a	2.88b	55.65b	286.48c
	75	46bc	43.5c	2.56a	39.12a	3.97a	1.12a	0.44a	3.08a	2.66b	51.42b	283.50c
	100	20d	76.47d	3.68a	28.82b	6.70b	1.15a	0.41a	2.66ab	2.44b	46.83b	102.04e
MgCl_2_	25	76a	10.58ab	2.31a	43.30a	3.38a	1.26a	0.62a	3.52a	2.55b	49.30b	462.58b
	50	72ab	15.30ab	2.58a	39.36a	3.98a	1.22a	0.46a	1.26bc	1.04c	19.67c	168.14d
	75	34c	60cd	2.50a	40.55a	3.98a	1.11a	0.46a	0.32cd	0.32d	6.15d	23.38f
	100	0e	100e	-	-	-	0b	-	0d	0d	0d	0g
Na_2_SO_4_	25	72ab	15.29b	3.06a	33.08ab	4.90ab	1.80a	0.54a	2.14b	2.62b	49.80b	306.62c
	50	57b	32.94c	3.12a	32.05ab	5.00b	1.64a	0.36a	0.74c	0.38cd	7.42d	64.44e
	75	42c	50.58c	3.57a	28.10b	5.82b	1.44a	0.33a	0.25d	0.36cd	6.76d	26.48f
	100	25d	70.58d	3.70a	27.04b	6.52b	0.80b	0.28a	0.14d	0.24d	4.64d	9.46g
K_2_SO_4_	25	74a	12.94ab	2.42a	40.75a	3.60a	1.14a	0.48a	3.28a	3.56b	67.88ab	501.02b
	50	42c	50.58c	2.84a	41.26a	4.62ab	1.50a	0.30a	1.60dc	1.34c	25.34c	133.42d
	75	30c	64.70cd	2.92a	35.30ab	4.84ab	1.30a	0.27a	0.50cd	0.48cd	9.28d	27.10f
	100	0e	100e	-	34.62ab	-	0b	-	0d	0d	0d	0g
MgSO_4_	25	85a	0a	2.61a	38.30a	4.08ab	1.32a	0.42a	3.42a	2.72b	51.70b	522.98b
	50	68b	20.02b	2.58a	41.14a	4.50ab	1.21a	0.48a	0.76c	0.90c	17.36cd	113.56e
	75	46bc	45.88c	2.61a	38.39a	5.06b	1.36a	0.38a	0.44cd	0.28d	5.28d	34.88f
	100	0e	100e	-	-	-	0b	-	0d	0d	0d	0g

SC: salt concentration; FGP: final germination percentage; DGP: decreasing germination percentage; MGT: mean germination time; GSP: germination speed; T_50_: time to reach 50% of final germination; Unc: uncertainty; Syn: synchrony; HL: hypocotyl length; RL: radicle length; STI: seedling tolerance index; SVI: seedling vigor index; NaCl: sodium chloride; KCl: potassium chloride; MgCl_2_: magnesium chloride; Na_2_SO_4_: sodium sulfate; K_2_SO_4_: potassium sulfate; MgSO_4_: magnesium sulfate. Values sharing the same letter are not significantly different (*p* ≤ 0.05, *Tukey*’s test).

**Table 4:** Seed germination patterns and seedling growth features of *M. communis* in response to different concentrations of chloride- and sulfate-based salts.

Salts	SC (mM)	FGP (%)	DGP (%)	MGT (Days)	GSP (%)	T_50_ (Days)	Unc	Syn (bit)	HL (cm)	RL (cm)	STI	SVI
Control	0	90a	0a	5.71a	17.52a	10.48a	2.22a	0.26a	1.50a	1.26a	100a	251.32a
NaCl	25	74b	17.52b	5.89a	17.02a	9.78a	2.30a	0.19a	1.22b	0.84b	70.92b	153.68b
	50	54c	41.17c	6.26a	15.96ab	11.06a	2.28a	0.17a	1.16b	0.72b	63.28bc	100.36c
	75	30d	67.72d	6.98ab	14.68bc	12.39a	2.04a	0.16a	0.82c	0.61b	55.06c	41.64d
	100	14e	83.72e	7.12ab	14.05bc	12.50a	1.25b	0.13a	0.60c	0.56b	52.38c	17.56f
KCl	25	84a	7.25a	6.2a	16.34ab	10.75a	2.48a	0.16a	1.94a	1.30a	111.80a	272.48a
	50	67b	26.35b	6.90ab	14.64bc	12.22a	2.61a	0.16a	1.50a	1.20a	103.26a	177.82b
	75	40c	54.42c	7.64b	13.10cd	14.04b	2.31a	0.13a	0.82c	0.90ab	77.98b	71.56d
	100	20d	78.06e	8.39b	11.97d	16.16b	1.44b	0.13a	0.50cd	0.50b	41.40c	20.66f
MgCl_2_	25	62bc	32.35c	5.86a	17.04a	10.85a	2.76a	0.18a	1.46a	1.14a	92.16ab	160.52b
	50	50c	45.59c	6.24a	16.08ab	11.06a	2.30a	0.18a	1.02b	0.88ab	68.75b	86.26cd
	75	34d	61.66d	7.40ab	13.52c	13.94ab	2.00ab	0.15a	0.90bc	0.72b	77.38b	62.58d
	100	22e	74.96d	9.21b	11.08d	18.00c	1.37b	0.13a	0.31d	0.48bc	43.15c	17.74f
Na_2_SO_4_	25	65b	27.80b	6.00a	16.68a	9.94a	2.40a	0.15a	0.96bc	0.78b	67.86b	113.82c
	50	52c	42.70c	6.72a	14.94bc	11.08a	2.24a	0.17a	0.82c	0.75b	65.48b	82.14c
	75	36d	60.14d	8.02b	12.42c	15.34b	2.30a	0.14a	0.58cd	0.50b	41.46c	39.38e
	100	18e	79.45de	9.16b	10.94d	17.55bc	1.52b	0.12a	0.25d	0.36c	32.74	11.12f
K_2_SO_4_	25	65b	27.92b	6.28a	15.92ab	11.06a	2.45a	0.16a	1.18ab	1.02ab	83.52ab	142.75b
	50	46c	48.62c	7.22ab	14.00bc	13.68ab	2.24a	0.15a	0.90bc	0.60b	53.58c	70.14d
	75	30d	66.20d	7.82b	12.86c	14.44b	2.10a	0.14a	0.66c	0.44bc	32.82d	33.42e
	100	20e	78.00e	8.52b	11.75d	16.16b	1.64b	0.13a	0.35d	0.32c	28.08d	13.6f
MgSO_4_	25	85a	5.80a	6.90ab	14.52bc	12.52a	2.74a	0.20a	1.40a	1.08a	96.42	213.24ab
	50	62bc	30.80bc	7.16ab	13.96bc	13.10ab	2.55a	0.13a	1.34a	0.92ab	77.68	141.95b
	75	36d	60.34d	7.80ab	12.98c	15.72b	2.20a	0.12a	1.30ab	0.84b	72.82	77.15d
	100	30d	67.65d	9.06b	11.28d	17.22bc	1.80ab	0.12a	0.86c	0.82b	68.25	49.64e

SC: salt concentration; FGP: final germination percentage; DGP: decreasing germination percentage; MGT: mean germination time; GSP: germination speed; T_50_: time to reach 50% of final germination; Unc: uncertainty; Syn: synchrony; HL: hypocotyl length; RL: radicle length; STI: seedling tolerance index; SVI: seedling vigor index. NaCl: sodium chloride; KCl: potassium chloride; MgCl_2_: magnesium chloride; Na_2_SO_4_: sodium sulfate; K_2_SO_4_: potassium sulfate; MgSO_4_: magnesium sulfate. Values sharing the same letter are not significantly different (*p* ≤ 0.05, *Tukey*’s test).

**Table 5:** List of the variables along with their meanings used for characterizing germination attributes.

Variables	Abbreviation	Definition
Final germination percentage	FGP	Percentage of seeds that successfully germinated
Mean germination time	MGT	Number of germinated seeds relative to non-germinated seeds at evaluation time
Germination speed coefficient	GSP	The rate of seed germination over a time interval
Germination uncertainty	Unc	Assessment of the variability in the distribution of germination frequencies
Germination synchrony	Syn	Degree of overlap in germination timing
Decreasing germination percentage	DGP	Expresses the difference in percentage between control and stressed seed lots
Time of 50% germination	T_50_	The average time required to achieve 50% germination

## Data Availability

All relevant data are within the paper.
